# Exercise modulation of tumour perfusion and hypoxia to improve radiotherapy response in prostate cancer

**DOI:** 10.1038/s41391-020-0245-z

**Published:** 2020-07-06

**Authors:** Oliver Schumacher, Daniel A. Galvão, Dennis R. Taaffe, Raphael Chee, Nigel Spry, Robert U. Newton

**Affiliations:** 1 Exercise Medicine Research Institute, Edith Cowan University, Joondalup, WA Australia; 2 School of Medical and Health Sciences, Edith Cowan University, Joondalup, WA Australia; 3 School of Human Movement and Nutrition Sciences, University of Queensland, Brisbane, QLD Australia; 4 GenesisCare, Joondalup, WA Australia

**Keywords:** Prostate cancer, Cancer therapy

## Abstract

**Background:**

An increasing number of studies indicate that exercise plays an important role in the overall care of prostate cancer (PCa) patients before, during and after treatment. Historically, research has focused on exercise as a modulator of physical function, psychosocial well-being as well as a countermeasure to cancer- and treatment-related adverse effects. However, recent studies reveal that exercise may also directly influence tumour physiology that could beneficially affect the response to radiotherapy.

**Methods:**

In this narrative review, we provide an overview of tumour vascular characteristics that limit the effect of radiation and establish a rationale for exercise as adjunct therapy during PCa radiotherapy. Further, we summarise the existing literature on exercise as a modulator of tumour perfusion and hypoxia and outline potential future research directions.

**Results:**

Preclinical research has shown that exercise can reduce intratumoral hypoxia—a major limiting factor in radiotherapy—by improving tumour perfusion and vascularisation. In addition, preliminary evidence suggests that exercise training can improve radiotherapy treatment outcomes by increasing natural killer cell infiltration in a murine PCa model.

**Conclusions:**

Exercise is a potentially promising adjunct therapy for men with PCa undergoing radiotherapy that may increase its effectiveness. However, exercise-induced tumour radiosensitisation remains to be confirmed in preclinical and clinical trials, as does the optimal exercise prescription to elicit such effects.

## Introduction

Radiotherapy is a cornerstone in the management of cancer and used to treat a myriad of malignancies, including prostate. Approximately 50% of all cancer patients receive radiotherapy during the course of their treatment for either curative or palliative purposes [1]. Moreover, ~40% of prostate cancer (PCa) patients aged 65 and above receive radiotherapy as their primary treatment [2]. Despite ever evolving technological advances that allow for more precise targeting of tumours, treatment resistance and subsequent disease recurrence pose a significant challenge in the field of radiation oncology [3–5].

Approximately 20–39% of patients with localised PCa treated with external beam radiotherapy experience local disease persistence and subsequent recurrence with an increased risk of developing distant metastases [6–8]. The hypoxic tumour microenvironment (TME) is recognised as a major limiting factor in the treatment of cancer and is an established prognostic marker that has been associated with treatment resistance as well as local disease persistence and recurrence [9, 10]. Moreover, hypoxia is a common feature of many solid tumours, including PCa [11].

While exercise has been associated with a reduced risk of PCa progression and mortality [12], little is known about the underlying biological mechanisms contributing to these benefits. It has been postulated that exercise may acutely reduce tumour hypoxia through increased perfusion of tumour tissue and that structural adaptations of the tumour vasculature in response to exercise training may potentially result in a less aggressive tumour and further enhance treatment response [13].

Given the significance of hypoxia in compromising cancer treatment, the purpose of this narrative review is to: (1) provide an overview of the characteristics of tumour vasculature and how they affect tumour hypoxia; (2) establish a rationale for exercise as adjunct therapy during cancer treatment; (3) summarise the existing literature on exercise as a modulator of tumour perfusion and hypoxia; and (4) outline potential future research directions.

## Tumour vasculature and hypoxia

Angiogenesis is a normal physiological process that occurs in situations like wound healing or in response to exercise training [14, 15]. In tumours, however, rapid proliferation of cells results in the formation of hypoxic areas and subsequent persistent local and unbalanced expression of pro- and anti-angiogenic factors leads to the formation of dysfunctional and structurally abnormal vascular networks, which are a characteristic feature of the TME [16]. These vascular networks are often very disorganised, tortuous and immature, which results in heterogeneous and variable tumour blood flow, further aggravating rather than alleviating hypoxia [17, 18]. Consequently, hypoxic and thus angiogenic signalling are sustained, further driving abnormal tumour vascularisation. In addition, tumour blood vessels are leakier than normal blood vessels, resulting in increased interstitial fluid pressure (IFP) [19]. Increased IFP can lead to sudden, intermittent perfusion changes as a result of collapsing blood vessels, which, in turn, limits oxygen delivery to the tumour, further worsening tumour hypoxia and decreasing treatment efficacy [20]. Moreover, increased vascular permeability plays a key role in metastasis by facilitating intravasation of cancer cells [21].

Hypoxia arising from vascular abnormalities can be differentiated into two forms—chronic and acute [22, 23]. Chronic hypoxia arises when the distance between blood vessels and tumour cells surpass the diffusion distance of oxygen. Chronic hypoxia is therefore also known as diffusion-limited hypoxia. When oxygen diffuses from blood vessels into the adjacent tissue it is consumed by the tumour cells closest to the perfused vessel, creating a gradient in the partial pressure of oxygen (*P*O_2_). The decrease in *P*O_2_ with increasing distance from the perfused vessel renders tumour cells further away from this vessel more hypoxic. Acute hypoxia, on the other hand, is caused by limited and varying perfusion of blood vessels supplying tumour cells (i.e. perfusion-limited hypoxia). As a result, all cells that are normally supplied by a now transiently compromised blood vessel are temporarily, or acutely, hypoxic. The reasons for acutely limited perfusion are numerous and include occlusion of vessels with blood or circulating tumour cells, collapse of vessels due to high IFP, and spontaneous vasoconstriction in normal tumour vessels that then affect downstream tumour vessels [22, 23]. In addition to poor tumour perfusion and oxygen diffusion, anaemia is a further major cause of tumour hypoxia (i.e. anaemic hypoxia) that results from cancer- and treatment-associated reduced oxygen transport capacity of the blood [24, 25].

With decreasing tissue oxygen levels, tumour cells become increasingly resistant to ionising radiation due to reduced availability of oxygen to stabilise DNA strand breaks caused by radiation and reactive oxygen species [23, 26]. In PCa, hypoxia is associated with a more aggressive tumour phenotype, increased metastasis, and patients with more hypoxic tumours have a poorer prognosis compared to patients with a less hypoxic TME [27]. Milosevic et al. [28] directly measured tumour hypoxia with a transrectal needle-electrode technique in 247 patients with localised PCa before radiotherapy and found that hypoxia was associated with early biochemical failure and local disease recurrence. Intrinsic markers of tumour hypoxia (i.e. hypoxia-inducible factor 1-alpha) have also been investigated and increased expression identified patients with shorter time to biochemical failure independent of clinical tumour stage, biopsy Gleason score, serum prostate-specific antigen concentration, and radiotherapy dose [29]. Targeting and reversing tumour hypoxia thus has been proposed as a potential mechanism to improve treatment response to radiotherapy and enhance survival [27].

Pharmacological interventions aimed at inducing vascular normalisation to improve treatment efficacy through increased drug delivery and tumour oxygenation have primarily focused on suppressing vascular endothelial growth factor (VEGF) signalling given its key role in angiogenesis [30]. Anti-angiogenic (or anti-VEGF) therapy, originally developed to disrupt a tumour’s blood supply and deprive it of oxygen and nutrients, has shown some promise in normalising tumour vasculature when given in low doses, but has thus far not been tested in prospective clinical trials to permit concrete conclusions [30, 31]. Furthermore, tumour vascular normalisation has a small therapeutic window, is complicated by tumour heterogeneity, and associated with significant side effects as well as malignant progression [32–35]. Hence, the use of pharmacological angiogenesis inhibitors has not gained wide clinical application. On the other hand, mild hyperthermia is increasingly being explored as a radiosensitiser for its effects on tumour vasculature and DNA repair mechanisms [36]. Relatively low temperatures of 39–42 °C have been shown to increase tumour perfusion, thus decreasing hypoxia and enhancing tumour radiosensitivity [36]. In a recent study using an orthotopic PCa mouse model, hyperthermia significantly slowed tumour growth and resulted in significantly smaller normalised nadir tumour volumes compared to radiotherapy without hyperthermia [37]. Similar to anti-VEGF therapy, though, the timing and dosing of hyperthermia to achieve the desired radiosensitising effect is complex and makes clinical application challenging [36].

## Exercise as adjunct therapy in cancer treatment

In the field of exercise oncology, i.e. the application of exercise as medicine in the oncology setting, there is consistent evidence that greater levels of physical activity, commonly defined as ‘any bodily movement produced by skeletal muscles that results in energy expenditure’ [38], are associated with a lower risk of developing certain types of cancer as well as decreased all-cause and cancer-specific mortality for patients diagnosed with breast, colorectal, or PCa [39]. For example, Kenfield et al. [40] found that men with PCa who did ≥3 h/week of vigorous physical activity had a 49% lower risk of all-cause mortality and a 61% lower risk of dying from PCa compared to men who did <1 h/week of vigorous physical activity. Furthermore, Richman et al. [41] reported that men with localised PCa who walked at a brisk pace for ≥3 h/week had a 57% lower risk of disease progression compared to men who walked at an easy pace for <3 h/week. This association between walking pace and reduced risk of cancer progression was independent of walking duration [41], suggesting that not only the volume of physical activity but also the intensity of physical activity is an important factor regarding clinical outcomes in PCa. It is important to note, however, that these findings are based on observational data and randomised controlled trials are currently investigating the impact of exercise on overall survival in patients with PCa as well as colon cancer [42, 43].

In addition to reduced risk of disease progression and mortality, physical activity and exercise, defined as ‘a subset of physical activity that is planned, structured, and repetitive and has as a final or an intermediate objective the improvement or maintenance of physical fitness’ [38], are increasingly being recognised as an important part of treatment during active therapy to improve or maintain physical function and quality of life in cancer patients [44]. Research has consistently demonstrated that exercise improves physical and mental health in men with PCa during and following completion of therapeutic interventions and can reduce the level of fatigue experienced by many patients [45–48]. Some studies have also indicated that exercise may help alleviate toxicities associated with treatment [49, 50].

**Fig. 1:**
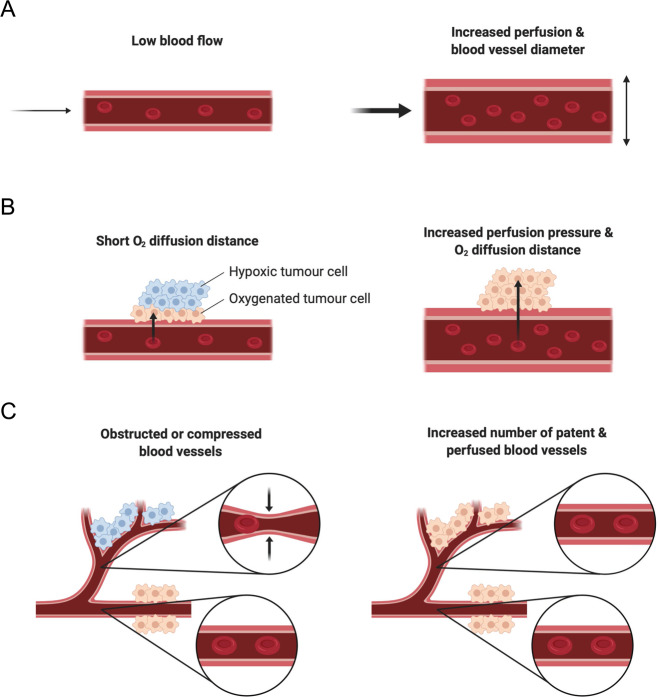
Potential tumour physiology at rest (left side) and during exercise (right side). **a** Acute exercise increases tumour blood flow and tumour vascular resistance is reduced. **b** Augmented tumour perfusion pressure as a result of increased tumour blood flow may lead to greater oxygen (O_2_) diffusion distance and thus reduce diffusion-limited hypoxic tumour areas. **c** Tumour vasculature is dysfunctional and structurally abnormal, resulting in heterogeneous and variable tumour blood flow. Exercise increases the area of tumour perfusion and therefore reduces perfusion-limited hypoxia. Created with BioRender.com.

**Fig. 2:**
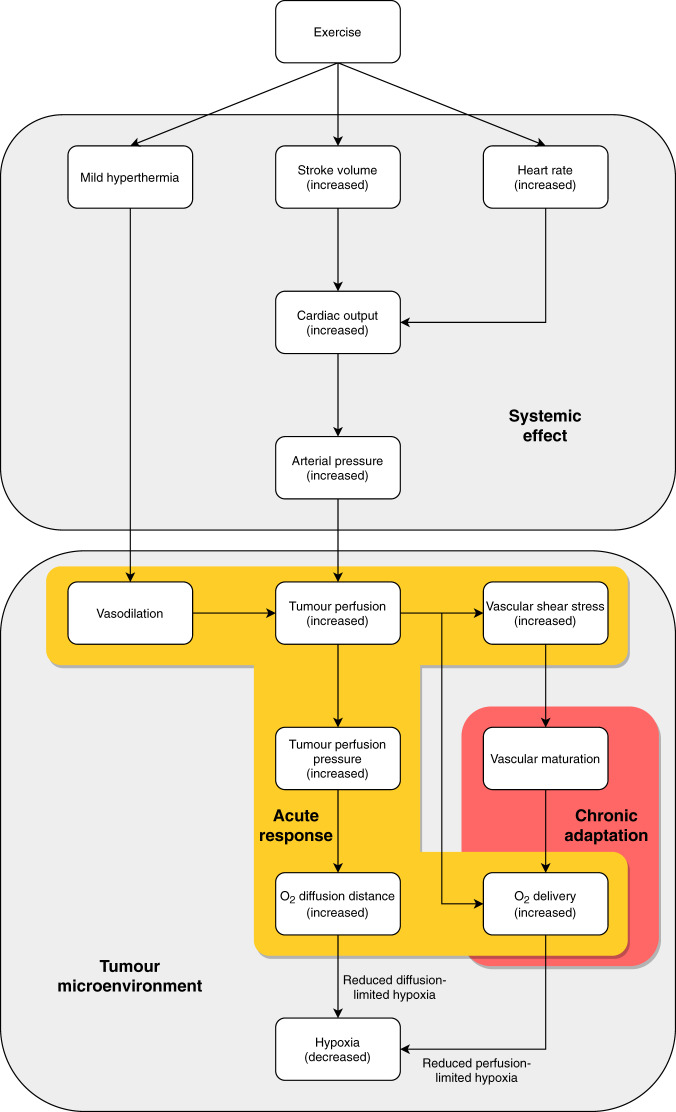
Potential mechanism of exercise-modulated tumour perfusion and hypoxia. Systemic effects of augmented cardiac output and increased arterial pressure resulting from mild hyperthermia and increased stroke volume and heart rate in response to acute exercise (top panel) may influence local physiological responses and vascular adaptation in the tumour microenvironment (bottom panel). Improved tumour perfusion, either as a result of increased systemic blood flow during exercise or as a result of vascular maturation, may enhance acute and chronic oxygenation of hypoxic tumour areas, thus resulting in improved radiotherapy efficacy.

More recently, the role of exercise as an adjunct therapy to enhance the effectiveness of conventional cancer treatments such as radiotherapy is gaining a strong interest for its effects of modulating tumour vasculature and oxygenation [51, 52]. Potential tumour physiological responses to exercise are shown in Fig. 1. However, mechanistic insight regarding the benefits of exercise on cancer biology is currently limited. Acute exercise bouts as well as exercise training are characterised by distinct physiological responses and result in specific immediate and chronic adaptations. It is well established that exercise leads to vascular adaptations in humans [53]. For example, exercise training improves oxygen delivery to peripheral tissues through vascular remodelling, namely angiogenesis and arteriogenesis [54, 55]. The same structural remodelling of the vasculature in response to exercise may also take place in tumours, thus potentially improving oxygenation in the same way it does in working muscles during and after exercise. Moreover, under acute exercise conditions, mechanical forces exerted on vessel walls increase with increasing exercise intensity and are thought to promote the development of functional mature vasculature [56, 57]. Translated to dysfunctional tumour vasculature, exercise may result in the development of more mature and less permeable blood vessels that function more normally. Furthermore, increased heart rate and stroke volume during exercise result in augmented cardiac output and increase systemic blood flow, potentially also enhancing perfusion of tumour tissue. Together with increased tumour blood flow as a result of vasodilation mediated by mild hyperthermia that may occur during exercise and increases in mean arterial pressure that could result in an increased oxygen diffusion distance within the tumour, there are several potential exercise-related mechanisms that may alleviate tumour hypoxia and therefore enhance treatment response to radiotherapy (Fig. 2).

## Exercise modulation of tumour perfusion and hypoxia

As detailed above, physical activity has been associated with a reduced risk of PCa progression and mortality [40, 41]. However, little is known about the underlying biological mechanisms that delay progression and improve survival in PCa patients. Potential mechanisms have been summarised by Galvão et al. [58] and include, among others, changes in tumour vascularisation that may permit better drug delivery and oxygenation of the tumour through improved perfusion [58]. Indeed, tumour vessel morphology has been associated with survival outcomes in men diagnosed with PCa. Mucci et al. [59] evaluated whether tumour microvessel morphology would predict PCa mortality among 572 men in the prostatectomy cohort of the Health Professionals Follow-up Study. While greater microvessel density (MVD) was associated with extraprostatic extension it was not a predictor of mortality in this analysis. However, the authors found that men with tumours containing the smallest (area and diameter) and most irregularly shaped vessels had a ~6 times and ~17 times higher likelihood of developing lethal PCa, respectively.

In a further investigation, also based on data from the Health Professionals Follow-up Study, Van Blarigan et al. [60] examined whether physical activity was associated with tumour vessel morphology in men with PCa. They found that men who reported a brisk walking pace of ≥4.8 km/h had larger, more regularly shaped blood vessels compared to men who reported walking at <4.8 km/h. However, there was no association between brisk walking and MVD, and the duration of physical activity (vigorous and non-vigorous) was not associated with tumour blood vessel size, shape or number [60].

In the following sections, we discuss the clinical and preclinical work that has been done to further shed light on potential mechanisms and how they might affect treatment outcomes. These sections are not intended to be an exhaustive review of the relevant literature that examine exercise as an adjunct therapy in cancer or a means to improve treatment outcomes. Rather, they are intended to provide an extensive overview of the efficacy of exercise to modulate tumour vasculature, perfusion and hypoxia as well as how these adaptations might translate to improved treatment outcomes. A literature search was conducted in Embase, MEDLINE and PubMed using the search terms exercise AND (cancer OR tumour) AND (perfusion OR blood flow OR hypoxia OR oxygen OR vascularity OR vascular OR vessels) as well as exercise AND prostate cancer AND (radiotherapy OR radiation), and last updated in February 2020.

### Preclinical studies

Aerobic exercise has been linked to a reduction in tumour incidence, growth and metastasis across a variety of preclinical tumour models. However, meaningful comparisons and conclusions from current evidence is limited due to considerable heterogeneity in the methods used across different studies [61]. It has been proposed that exercise can directly impact tumour physiology and improve anti-cancer treatment efficacy, thus highlighting the potential of exercise to control disease progression [62]. One important mechanism by which exercise may govern these processes is modulation of tumour vascularisation and perfusion [63].

#### Acute exercise responses

Compared with short- as well as long-term exercise training interventions, few studies have investigated how an acute bout of exercise may affect tumour physiology (Table 1). Using a preclinical PCa model, McCullough et al. [64] published the first study to investigate tumour blood flow in vivo during exercise. The authors demonstrated that compared to resting conditions, exercise comprised of forced treadmill running significantly increased tumour blood flow by ~200% as well as the number of perfused blood vessels, with hypoxia decreased by ~50%. These results were later replicated by Garcia et al. [65], who used a similar exercise protocol in a PCa model and demonstrated that exercise increased tumour blood flow by ~180% compared to resting conditions. Interestingly, this response was only observed in orthotopic tumours mimicking the physiological conditions of primary cancers. Ectopic tumours, which could be regarded as metastatic lesions, showed a decrease in blood flow of ~25% in response to exercise.

**Table 1:** Acute effects of exercise on tumour perfusion and hypoxia.

Author (reference)	Tumour model	Intervention	Exercise	Results	Treatment outcome
McCullough et al. [64]	Male Copenhagen rats with Dunning R3327-MatLyLu prostate adenocarcinoma	Exercise vs. rest	Forced treadmill running at 15 m/min (10° incline) for 5 min (perfusion measurements) or 60 min (hypoxia measurements)	↑ perfusion (~200%) vs. rest; ↓ vascular resistance (~65%) vs. rest; ↑ average number of patent vessels vs. rest; ↓ hypoxia (~50%) vs. rest	NA
Miller et al. [66]	Female C57BL6/KaLwRij mice with 5TGM1 myeloma tumours and female BALB/c mice with MPC11 myeloma tumours	Exercise vs. anaesthesia	Forced treadmill running at up to 20 m/min for 20 min	↑ radionuclide uptake vs. anaesthesia; ↑ homogeneity of infection distribution vs. anaesthesia; ↓ average infection voids vs. anaesthesia	↑ survival vs. anaesthesia; ↓ disease progression vs. anaesthesia; ↑ tumour response vs. anaesthesia; ↑ tumour growth inhibition vs. anaesthesia
Garcia et al. [65]	Male Copenhagen rats with Dunning R3327-MatLyLu prostate adenocarcinoma	Exercise vs. rest	Forced treadmill running at 15 m/min for 5 min	↑ perfusion (~181%) in orthotopic tumour vs. rest; ↓ perfusion (~25%) in ectopic tumour vs. rest	NA
Schadler et al. [67]	C57BL/6J mice with B16F10 melanoma tumours	Exercise vs. no exercise (control)	Forced treadmill running at 10 m/min for 45 min	↔ doxorubicin levels in tumours vs. control	NA
Lønbro et al. [68] (Abstract)	Female CDF1 mice with C3H mammary carcinoma	Exercise vs. no exercise (control)	Forced treadmill running at either 6 m/min (low), 12 m/min (moderate) or 18 m/min (high intensity) for 30 min	↓ hypoxia for high intensity vs. control; ↔ hypoxia for low and moderate intensity vs. no exercise	NA

↑ increased, ↓ decreased, ↔ unchanged/no difference, *NA* not assessed.

Miller et al. [66] and Schadler et al. [67] used radionuclide uptake and intratumoral doxorubicin levels as surrogate markers for tumour perfusion, respectively. Whereas Miller et al. [66] found an increased concentration of radiotracers with high-intensity exercise compared to a hypotensive low-perfusion state induced by anaesthesia, Schadler et al. [67] found no difference in doxorubicin levels after low-intensity exercise compared to mice not undergoing exercise. Despite exercising for more than twice the amount of time (45 vs. 20 min), the mice in the experiment by Schadler et al. [67] only exercised at half the intensity (10 vs. 20 m/min), which likely resulted in overall lower mean arterial pressure compared to the experiment by Miller et al. [66] and therefore could have resulted in lower tumour perfusion pressure and hence lower tumour blood flow. Indeed, preliminary data from Lønbro et al. [68] suggest that it is only high-intensity exercise and not low- or moderate-intensity exercise that results in decreased intratumoral hypoxia.

The studies by McCullough et al. [64] and Garcia et al. [65] both also investigated in vitro vascular responsiveness to norepinephrine. In tumour arterioles, vasoconstriction was blunted by approximately 95% compared with control prostate vessels [64], and although there was no difference in vasoreactivity between orthotropic and ectopic tumours, vasoconstriction was severely reduced compared with host prostate tissue from healthy control animals [65]. This blunted vascular responsiveness is potentially what allows tumour perfusion to be either maintained or increased during exercise, when blood would otherwise normally be diverted away from internal organs to working muscles. Importantly, the resulting increase in tumour blood flow may play a critical role in improving oxygenation for radiotherapy as well as tumour-targeting drug delivery. Additionally, it has been proposed that acute systemic effects of exercise in combination with increased tumour perfusion may contribute to slower tumour progression [62]. However, it is currently unknown for how long after exercise these changes in tumour perfusion last and hence when the optimal time would be to exercise in relation to treatment, or at which point there is no added benefit of exercise. Furthermore, it is unclear what exercise prescription in terms of intensity and duration would provide the most potent stimulus that is both feasible and produces a clinically meaningful response.

#### Adaptations to exercise training

Several animal model studies, predominantly in lymphoma, breast and PCa, that investigated the effect of exercise training on tumour physiology, including vascular function and hypoxia, also assessed tumour growth. In studies by Jones et al. [69, 70], tumour growth rate was comparable between exercise groups who had voluntary access to a running wheel and non-exercising control groups. On the other hand, exercise significantly reduced tumour growth in a murine breast cancer model [71]. Buss and Dachs [72] also found that voluntary wheel running slowed tumour growth rate in hyperlipidemic mice. However, this was only the case for mice with high muscular cytochrome *c* oxidase subunit IV (COX-IV) expression (a biomarker used for the level of exercise), suggesting that only mice with higher exercise volumes experienced a reduction in tumour growth rate [72]. Moreover, exercise appeared to suppress the formation and growth of metastases in exercising animals [69, 72]. The anti-metastatic effect of exercise may be linked to its ability to alter vascular permeability through modulation of redox-sensitive small GTPase activity, thereby preventing cancer cells from easily crossing the vessel wall, making it more difficult to establish metastatic sites [73]. Indeed, studies by Wolff et al. showed that running distance was inversely correlated with activation of the redox-sensitive small GTPase Rho [74], and that exercise maintained blood–brain barrier integrity, thus preventing tumour cells from extravasating into the brain [75].

An overview of the studies investigating the impact of exercise training on tumour vasculature in an attempt to alleviate hypoxia and improve treatment response are presented in Table 2. Both short-term and long-term exercise training, in the form of voluntary wheel running or forced treadmill running, appear to improve intratumoral vasculature. However, while changes in vessel number and size as well as MVD are inconsistent, findings of increased vessel maturity and function that allow for tumours to be perfused more uniformly and thus increase microvascular *P*O_2_ are more conclusive. Schadler et al. [67] postulated that increased shear stress exerted on endothelial cells during aerobic exercise plays an important role in the remodelling of tumour blood vessels, allowing for greater blood flow to the tumour. In addition, McCullough et al. [76] found that temporal variations in microvascular *P*O_2_ were reduced in exercise-trained animals compared to sedentary control rats in an orthotopic PCa model. Studies have also shown that the number of patent and perfused vessels increased after exercise training, resulting in a larger percentage and less heterogeneously perfused tumour area [67, 69, 70, 77]. These changes in vascularisation and improvements in perfusion were accompanied by a reduction in tumour hypoxia [71, 72, 76, 78, 79], and mice with high COX-IV expression had significantly lower levels of hypoxia compared to mice with low levels of COX-IV expression [72].

**Table 2:** Effects of exercise training on tumour vasculature, hypoxia and treatment outcomes in preclinical and clinical studies.

Author (reference)	Tumour model/patient population	Intervention	Exercise	Results	Treatment outcome
**Preclinical studies**					
Zielinski et al. [86]	Female BALB/cByJ mice with EL-4 lymphoma tumours	Exercise vs. sedentary control	Forced treadmill running at gradually increasing speeds (20–40 m/min) at a 5% grade for 3 h or until volitional fatigue on every day for 5–14 days	↓ blood vessel density (25–50%) vs. control; ↓ blood vessel density over time in both groups	NA
Verma et al. [79]	Male BALB/c with Dalton’s lymphoma tumours	Exercise vs. no exercise (control)	Forced treadmill running at gradually increasing speeds up to 17 m/min for 30 or 2 × 30 min (with 15 min rest) for 10 days	↓ size and branching of blood vessels vs. control; ↑ oxygenation vs. control	NA
Jones et al. [70]	Athymic homozygous female mice with MDA-MB-231 mammary adenocarcinoma	Exercise vs. sedentary control	Voluntary wheel running (i.e. intermittent periods of high-intensity, short-duration exercise with low resistance) for 44 ± 3 days	↑ mean number of perfused vessels vs. control; ↑ percentage of perfused total tumour area vs. control; ↑ hypoxia of total tumour area vs. control	NA
Jones et al. [69]	C57BL/6 male mice with TRAMP-C1 prostate adenocarcinoma	Exercise vs. sedentary control	Voluntary wheel running (i.e. intermittent periods of high-intensity, short-duration exercise with low resistance) for 8 weeks	↑ hypoxia vs. control; ↑ perfusion vs. control; ↑ perfusion homogeneity vs. control; ↑ vascularisation	NA
McCullough et al. [76]	Male Copenhagen and Nude rats with Dunning R-3327 AT1 prostate adenocarcinoma	Exercise vs. sedentary control	Forced treadmill running at 15 m/min (15° incline) for 60 min/day for 5–7 weeks	↓ microvascular *P*O_2_ variance during measurement vs. control; ↑ microvascular *P*O_2_ (~100%) vs. control; ↔ number of patent vessels; ↓ hypoxia vs. control; ↓ vasoconstriction vs. non-tumour-bearing sedentary control; ↔ vessel contractile function vs. tumour-bearing sedentary control	NA
Betof et al. [71]	Female BALB/c mice with 4T1 mammary carcinoma and C57BL/6 mice with E0771 mammary adenocarcinoma	Exercise vs. sedentary control	Voluntary wheel running (i.e. intermittent periods of high-intensity, short-duration exercise with low resistance) for 18 days	↑ MVD vs. control; ↑ vessel maturity vs. control; ↑ viable tumour area containing pericyte-covered vessels and vessel area covered by pericytes vs. control; ↑ perfusion vs. control; ↓ hypoxia (~50%) vs. control	↑ apoptosis vs. control; ↓ tumour growth rate with exercise + chemotherapy vs. chemotherapy alone; ↔ tumour growth rate with exercise only vs. chemotherapy only
Faustino-Rocha et al. [87]	Female Sprague-Dawley rats with mammary tumours induced by *N*- methyl-*N*-nitrosourea	Exercise vs. sedentary control	Forced treadmill running at 20 m/min for 60 min/day for 5 days/week for 35 weeks	↑ MVD vs. control	NA
Faustino-Rocha et al. [88]	As above (see Faustino-Rocha et al. [87])	As above (see Faustino-Rocha et al. [87])	As above (see Faustino-Rocha et al. [87])	↔ vascularisation vs. control	NA
Schadler et al. [67]	C57BL/6J mice with B16F10 melanoma tumours or PDAC-4662 pancreatic ductal adenocarcinoma	Exercise vs. no exercise (control)	Forced treadmill running at 10 m/min (for melanoma tumour-bearing mice) or 12 m/min (for PDAC tumour-bearing mice) for 45 min/day for 5 days/week for up to 21 days	↔ MVD vs. control; ↑ number of long vessels vs. control; ↑ average vessel length vs. control; ↑ number of visible lumens vs. control; ↑ lectin-positive (functional) vessels (24%) vs. control	↓ tumour growth with exercise + chemotherapy vs. chemotherapy alone; ↑ DNA damage with exercise + chemotherapy vs. chemotherapy alone
Buss et al. [72]	C57BL/6 ApoE^−/−^ mice with E0771 mammary adenocarcinoma	High vs. low muscular COX-IV expression	Voluntary wheel running	↔ perfusion vs. low muscular COX-IV expression; ↓ hypoxia vs. low muscular COX-IV expression	NA
Morrell et al. [78]	Male Nude mice with A673 or TC71 Ewing sarcoma	Exercise vs. no exercise (control)	Forced treadmill running at 12 m/min for 45 min/day for 5 consecutive days/week for 2 weeks	↔ tumour vessel morphology (open lumens, total vessels, MVD, number of elongated vessels) vs. control; ↓ hyperpermeability vs. control; ↓ hypoxia vs. control; ↑ doxorubicin levels in tumours vs. control	↑ tumour growth inhibition with exercise + doxorubicin vs. doxorubicin alone
Florez Bedoya et al. [77]	Female Nude mice with PDAC patient-derived xenograft	Exercise vs. no exercise (control)	Forced treadmill running at 12 m/min for 45 min/day for 5 days/weeks for 4 weeks	↑ tumour vascularity (number of vessels, open lumens, elongated vessels) vs. control; ↑ percentage of lectin-positive (functional) vessels vs. control (*p* = ns)	↓ time to tumour regression (no palpable tumour) with exercise + gemcitabine vs. gemcitabine alone; ↑ time to tumour recurrence with exercise + gemcitabine vs. gemcitabine alone
Dufresne et al. [80]	Male athymic nude mice with PPC-1 prostate tumours	Exercise vs. radiotherapy vs. exercise plus radiotherapy vs. no exercise (control)	Forced treadmill running at 18 m/min (10% incline) with gradually increasing duration (25–60 min/day) for 5 days/week for 2 weeks	↑ vascularisation with radiotherapy and exercise plus radiotherapy vs. control and exercise only; ↔ vascularisation with exercise only vs. control; ↔ vascularisation with radiotherapy plus exercise vs. radiotherapy only; ↑ natural killer cell infiltration and activation with exercise plus radiotherapy and radiotherapy alone vs. exercise only and control; ↑ natural killer cell infiltration and activation with exercise plus radiotherapy vs. radiotherapy alone	↓ tumour volume and weight with exercise, radiotherapy and radiotherapy plus exercise vs. control; ↓ tumour cell proliferation with exercise, radiotherapy and radiotherapy plus exercise vs. control; ↑ apoptosis with exercise plus radiotherapy and radiotherapy alone vs. exercise only and control; ↑ apoptosis with exercise plus radiotherapy vs. radiotherapy alone
**Clinical studies**					
Jones et al. [81]	Women with operable stage II/III breast cancer (*n* = 20) receiving first-line neoadjuvant chemotherapy	Neoadjuvant chemotherapy vs. neoadjuvant chemotherapy plus aerobic exercise training	Cycling 3 days/week at 60–100% of *V*O_2_ peak for 30–45 min/session on a stationary cycle ergometer	↓ tumour blood flow (38%; exercise group); ↔ MVD (between groups); ↔ tumour hypoxia (between groups)	NA
Florez Bedoya et al. [77]	PDAC patients (*n* = 36) receiving preoperative chemotherapy or chemoradiotherapy	Exercise during preoperative chemo- or chemoradiotherapy vs. historical controls (no exercise)	Home-based, moderate-intensity exercise for at least 120 min/week (prescribed; 60 min aerobic exercise and 60 min strengthening exercises)	↑ number of vessels (100%) vs. control; ↑ MVD vs. control; ↑ number of elongated vessels vs. control; ↑ number of open lumens vs. control	NA

↑ increased, ↓ decreased, ↔ unchanged/no difference, *COX-IV* cytochrome *c* oxidase subunit 4, *DNA* deoxyribonucleic acid, *MAP* mean arterial pressure, *MVD* microvessel density, *NA* not assessed, *PDAC* pancreatic ductal adenocarcinoma, *P*O_2_, partial pressure of oxygen, *VO*_*2*_ *peak* volume of peak oxygen uptake.

#### Effect of exercise on radiotherapy treatment response

To date, only one study has investigated the effect of exercise in combination with radiotherapy on treatment outcomes [80]. Dufresne et al. injected human PCa PPC-1 cells subcutaneously into male athymic nude mice and subjected them to exercise 5 days/week for 25–60 min/day over 2 weeks in combination with a total of 20 Gray (Gy) fractionated into four doses of 5 Gy each. Mice were randomised to either no exercise (control), exercise only, radiotherapy only, or exercise plus radiotherapy. In contrast to previous studies, exercise alone did not increase tumour vascularisation; however, radiotherapy (with or without exercise) did. Radiotherapy further caused an increase in natural killer cell infiltration and activity that resulted in increased tumour cell apoptosis. Interestingly, this response was potentiated when exercise was combined with radiotherapy [80]. These results are exciting and provide preliminary preclinical evidence on the application of exercise as an adjunct therapy during radiotherapy in PCa.

### Clinical studies

Despite the large number of clinical studies in the field of exercise oncology and although preclinical studies have produced promising results, very few clinical studies have actually investigated the effect of either an acute bout of exercise or prolonged exercise training on tumour vasculature, perfusion and hypoxia in humans. To our knowledge, only two studies have been conducted in a clinical population [77, 81] (Table 2). Jones et al. [81] performed a pilot study where they allocated 20 women with operable breast cancer receiving neoadjuvant chemotherapy to either chemotherapy alone or chemotherapy plus aerobic exercise training, consisting of thrice weekly cycling on a stationary ergometer at 60–100% of peak oxygen consumption for 30–45 min/session. At baseline and after 9 weeks of the intervention (i.e. after three cycles of chemotherapy), tumour blood flow was assessed using ^15^O-labelled water positron emission tomography (PET). In the exercise group, tumour blood flow decreased by an average of 38%. However, the analysis was limited by the fact that pre/post PET scans were only available for five women in the exercise group (for two women in the control group for whom pre/post PET scans were available data were not reported). Further immunohistochemical analysis of tumour biopsies at the same time point (i.e. after 9 weeks of the intervention) revealed no difference between groups in relation to markers of hypoxia and MVD. However, similar to the PET assessment, tissue samples were only available for a small number of patients due to pathologic complete response rates to chemotherapy treatment.

In contrast, Florez Bedoya et al. [77] assessed tumour vessel morphology in tissue samples from 23 pancreatic ductal adenocarcinoma patients participating in an exercise intervention during preoperative chemo- or chemoradiation therapy and compared them to 13 historical controls (i.e. tumour samples from patients not undergoing exercise prior to surgery). The authors reported that 2 h/week of prescribed home-based exercise training resulted in remodelling of the tumour vasculature. Specifically, the total number of vessels, the number of elongated vessels, MVD as well as the number of open vessel lumens were all increased in samples from the exercise group compared with control samples. In theory, these vascular adaptations could result in improved chemotherapy and/or oxygen delivery to the tumour and hence improve clinical outcomes. However, further clinical studies are warranted to confirm these results and evaluate the effects of exercise-induced vascular remodelling on treatment efficacy as well as long-term outcomes.

Neoadjuvant and pre-surgical settings provide an excellent opportunity to study some of these outcomes. Indeed, several clinical trials are currently ongoing or have recently been completed and are pending publication of results, which will add to this body of knowledge. The majority of these registered studies are being conducted in PCa patients, either investigating vascular adaptations in response to exercise training prior to prostatectomy and subsequently analysing resected tumour tissue samples for tumour vessel morphology and hypoxic tumour area as well as intratumoral natural killer cell infiltration (ClinicalTrials.gov Identifier: NCT02954783 and NCT03365076), or investigating changes in tumour perfusion and hypoxia in response to acute exercise exposure using either magnetic resonance imaging or by pathological analysis of tumour tissue samples excised during radical prostatectomy (ANZCTR Registration number: ACTRN12620000432910; ClinicalTrials.gov Identifier: NCT03675529). These studies will add further information regarding exercise as a potential modulator of the TME in clinical populations and provide insight into mechanistic pathways.

## Implications for future research

Whereas initial clinical research shows mixed results, preclinical studies indicate that exercise may beneficially alter tumour physiology. In addition, one study in a PCa mouse model found that exercise increases the efficiency of radiotherapy, resulting in decreased tumour growth. These findings are promising and should encourage further investigation into this novel area of exercise and PCa research. However, given the paucity of research to date it is unclear what exercise prescription would deliver the desired outcome. Furthermore, specific tumour characteristics such as the initial degree of vascularity and metabolism will likely also influence outcome measures such as hypoxia and should be taken into consideration when designing future studies.

From a physiological perspective, the FITT principles of training, i.e. frequency, intensity, time (duration) and type of exercise should be considered as a starting point to formalise structured research questions. Different exercise intensities and types (e.g. aerobic vs. resistance training) are associated with distinct biochemical as well as physiological adaptations that may differentially affect tumour physiology. Furthermore, not all frequency schedules will likely be feasible for a specific exercise intensity (e.g. daily exercise at high intensity) and carefully considered periodisation schedules will need to be employed to maintain exercise tolerability during treatment [44, 82].

A concept that could be particularly interesting in this setting and patient population is the ‘minimal dose approach’ to exercise [83]. Considering the busy appointment schedule of patients, the minimal effective ‘dose’ of exercise (in terms of frequency, intensity and duration/time, or volume) would be extremely valuable to minimise patient burden. Hence, establishing or disproving dose–response relationships as well as threshold or ceiling effects will be essential to facilitate research translation into clinical practice. Similar to pharmaceutical drugs, exercise will likely also have a therapeutic window, where a certain minimal ‘dose’ is required to produce a desired effect and an upper tolerable ‘dose’ that will determine what is actually feasible for patients to safely accomplish.

In addition to the FITT principles of training, the timing of exercise in relation to treatment is likely to also be of importance. To achieve an oxygen enhancement effect for radiotherapy, the timing of exercise (i.e. pre-radiation) seems crucial, given that cellular oxygen is required at the time of radiation. Also, a distinction should be made between acute physiological changes and structural vascular adaptations that may aid treatment delivery and improve efficacy, including the time course in which these will likely occur.

From a clinical point of view, safety and efficacy trials in human populations are warranted. Although exercise is not a drug, it does drive a myriad of endogenous molecules to be produced that have influence locally and systemically as well as the effects of physical loading on vessels and other structures causing acute and chronic effects and adaptations. Therefore, it has been proposed that combining exercise with conventional cancer treatments should be held to the same standards as other combination treatments when evaluating treatment outcomes [84, 85]. Furthermore, logistical aspects that may affect the biological response also need to be taken into account. For example, studies in PCa patients investigating exercise in combination with radiotherapy may consider the timing and quantity of fluid intake before radiotherapy sessions, as gastrointestinal passage time and thus urine production may be affected by certain exercise durations and intensities. This may affect the positioning of the tumour mass due to fluctuating bladder volumes and ultimately lead to less radiation dose being delivered to the tumour and cause more toxicity to surrounding tissues.

Despite these potential challenges of implementing such trials, they will be a valuable addition to our understanding of the interaction between exercise and the TME and, if shown to be effective, will have clinical implications beyond the PCa population. However, men with PCa may particularly benefit from these developments, as initial observations appear to indicate that exercise of at least moderate intensity is required to elicit beneficial effects (i.e. increased tumour perfusion and subsequently reduced hypoxia). Men with PCa are usually relatively asymptomatic with respect to their disease and probably have a higher exercise capacity than many other cancer groups, such as pancreatic cancer patients, thus allowing them to exercise at an intensity to gain a benefit. Furthermore, men awaiting radical prostatectomy represent an ideal cohort to study these interactions as it allows researchers to directly study the impact of exercise on the tumour without the interference of neoadjuvant treatments and without having patients undergo additional invasive procedures to acquire tissue samples required to investigate potential mechanisms.

Collectively, further research in this area will add to the understanding of the underlying biological mechanisms of combination treatments involving exercise and address safety as well as dosing considerations important to both patients and clinicians. As a first important step, however, studies should address proof-of-concept outcomes such as short- and/or long-term reductions in tumour hypoxia as well as acute and chronic tumour perfusion changes as a result of exercise. We suggest these studies should incorporate non-invasive functional imaging modalities and immunohistochemical techniques to monitor tumour responses and pinpoint molecular mechanisms, with closely defined and reported patient populations and tumour characteristics as well as exercise prescription parameters to then develop more targeted interventions. Potentially, the findings of these studies will change best practice in radiation oncology with patients completing brief exercise bouts immediately (or shortly) before radiation doses, which may result in increased therapy success and a greater rate of cancer cure or delayed disease progression.
